# Longitudinal Follow-Up of microRNA Expression During Hepatitis E Virus Infection in Immunocompromised Hosts

**DOI:** 10.3390/biom16060799

**Published:** 2026-05-28

**Authors:** Nancy León-Janampa, Denys Brand, Julien Marlet

**Affiliations:** 1 INSERM U1259 MAVIVHe, Tours University and Tours University Hospital, 37000 Tours, France; 2 Department of Bacteriology-Virology-Hygiene, Tours University Hospital, 37044 Tours, France

**Keywords:** immunocompromised model, acute hepatitis E, chronic hepatitis E, microRNAs, longitudinal study

## Abstract

Hepatitis E virus (HEV) is a zoonotic cause of acute viral hepatitis that can become chronic in immunocompromised patients. Recently, host microRNAs (miRNAs) miR-122-5p, miR-125b-5p, miR-99a-5p, and miR-192-5p have been associated with acute HEV infection and with chronic HEV infection. Yet, the expression levels of miRNAs during the early phase of HEV infection remained unexplored. In this context, we aimed to identify changes in miRNA profiles occurring between the diagnosis of HEV infection and HEV cure in immunocompromised patients and in a pig model. We retrospectively included 9 immunocompromised patients with HEV infection (5 chronic and 4 resolutive) and 10 immunocompromised pigs (6 HEV-infected and 4 controls). RNAseq was retrospectively performed on pig samples. MiRNA expression was quantified by qPCR on pig and human samples.: Small RNA sequencing and targeted qPCR analyses revealed a pronounced, stage-dependent remodeling of miRNA expression during HEV infection. In patients, viral clearance was associated with early upregulation of miR-99a-5p, miR-122-5p, miR-125b-5p, and miR-194-5p, whereas progression to chronic infection was characterized by sustained downregulation of miR-23a-3p, miR-27a-3p, miR-106a-5p, miR-221-3p, and miR-223-3p. Likewise, a correlation was observed between the dysregulation of miR-181a-5p, miR-425-5p, and let-7b-5p with viral load (HEV RNA), while miR-27a-3p and miR-106a-5p were associated with ALT levels. In pigs, miR-122-5p was upregulated 10 weeks p.i., while 10 miRNAs (miR-16, miR-20a-5p, miR-23a-3p, miR-27a, miR-29a-3p, miR-140-5p, miR-181a, miR-191, miR-423-5p, and miR-99a-5p) were downregulated 11 weeks p.i. MiR-23a-3p, miR-27a, miR-99a-5p, miR-181a, and miR-425-5p were associated with HEV infection in both humans and pigs. Persistent HEV infection in immunocompromised patients and in a pig model showed partial overlap in miRNA responses. Our findings identified circulating miRNAs as stage-specific molecular markers of HEV infection in immunocompromised hosts. MiRNAs could contribute to early stratification of high-risk patients, improving outcomes for vulnerable patients facing this emerging viral disease.

## 1. Introduction

Hepatitis E virus (HEV) is a zoonotic cause of acute viral hepatitis that can become chronic in immunocompromised patients (≥3 months of infection). In vulnerable populations, chronic HEV infection may lead to rapid fibrosis and graft rejection, yet predictors of chronic outcome are lacking. Recent evidence implicates host microRNAs (miRNA or miR) as key regulators of HEV infection [1,2,3]. Host miRNAs are small noncoding RNAs that modulate gene expression and immune responses. During HEV infection, several liver-enriched miRNAs have been shown to directly enhance viral replication. For example, the addition of miR-122 (the most abundant liver miRNA) to hepatoma cultures greatly enhanced HEV replication, whereas miR-122 inhibitors suppressed viral replication [4]. This effect was mediated by direct binding of miR-122 to a conserved site in the HEV ORF1 polymerase region [4]. Similarly, miR-214 has been shown to promote HEV replication. Patil et al. demonstrated that miR-214 directly interacts with HEV RNA to boost viral genome translation and ORF2 protein production [5]. Elevated miR-214 also downregulates host protein C (a thrombin inhibitor), thereby increasing intracellular thrombin activity to facilitate HEV polyprotein cleavage [5]. Thus, host miR-122 and miR-214 function as proviral factors in HEV infection, fine-tuning both viral gene expression and the host inflammatory response [4,5]. In particular, miR-122 is known to repress negative regulators of interferon (IFN) signaling in hepatocytes, linking miR-122 levels to innate immune activation during infection.

Beyond these mechanistic studies, clinical profiling in humans has begun to reveal specific miRNA signatures in immunocompetent patients with acute HEV infection and immunocompromised patients with chronic HEV infection. Harms et al. found that several liver-enriched miRNAs (including miR-122-5p, miR-125b-5p, miR-99a-5p, and miR-192-5p) were significantly upregulated in HEV-infected patients compared to uninfected controls, and their levels differed between acute and chronic cases. In particular, miR-125b-5p and miR-99a-5p were highly elevated in both acute and chronic HEV, whereas miR-192-5p was increased only in chronic HEV, and miR-125b-5p was actually decreased in chronic versus acute infection. These findings suggested that a panel of miRNAs (miR-125b-5p, miR-192-5p, miR-99a-5p) could be associated with HEV infection in immunocompromised patients. HEV infection in immunocompromised patients is at risk of chronic hepatitis E (60%). It would be relevant to determine whether miRNA expression profiles during acute HEV infection in immunocompromised patients can be used to predict which patients will progress to chronic HEV infection.

Exploration of these early phases of HEV infection requires relevant models, such as animal models. Recently, we developed a clinically relevant pig model of chronic HEV infection by infecting tacrolimus-immunosuppressed pigs with HEV-3 [6]. This model recapitulated key features of human chronic HEV, persistent viremia, mild liver inflammation and fibrosis, and downregulated hepatic IFN responses, highlighting pigs as a powerful platform to study HEV pathogenesis [6]. Large white pigs are the natural reservoir of HEV genotypes 3 and 4 and share immunological similarities with humans, making them highly relevant for translational studies. However, to our knowledge, no studies have yet profiled host circulating miRNAs in immunocompromised HEV-infected pigs or any animal model. Animal models offer the opportunity for controlled, longitudinal sampling.

We hypothesized that longitudinal follow-up of serum miRNA expression profiles in immunocompromised hosts could lead to the identification of miRNA profiles associated with the pathogenesis of chronic hepatitis E, particularly during progression to chronicity. To this end, we first performed a high-throughput screening by RNAseq of miRNAs in serum samples collected 10 weeks p.i. (post-infection) during persistent HEV infection in a pig model [6]. This led to the identification of a subset of relevant miRNAs. These were further explored by qPCR in pigs and humans during the acute and chronic phases of HEV infection. Paired samples collected after HEV cure in patients (i.e., undetectable HEV RNA in plasma and feces) or before inoculation in pigs were used as controls to reduce inter-individual variability due to patient disease or immunosuppressive drugs [7,8,9].

## 2. Materials and Methods

### 2.1. Pig Samples

We previously published a pig model of persistent HEV-3 infection [6]. Twenty HEV-free Large White pigs were separated into a control group (*n* = 5), an immunocompromised non-infected group (*n* = 5), and an immunocompromised infected group (*n* = 10). Pigs were immunosuppressed with tacrolimus (Astellas Pharma Inc., Tokyo, Japan), mycophenolate mofetil (Sanofi, Paris, France), and prednisolone (Sanofi), with dose adjustments based on trough levels and clinical signs, based on kidney-transplant immunosuppression guidelines [10]. After two weeks of treatment, infected pigs were intravenously inoculated with 1 mL of a HEV-3f inoculum (patient feces) and monitored weekly for 11 weeks. For the present study, we retrospectively included 10 immunocompromised pigs (6 infected and 4 controls). From these, 31 serum samples were collected at 0 (i.e., before infection, *n* = 6), 4 (*n* = 4), 8 (*n* = 2), 10 (*n* = 6), and 11 (*n* = 6) weeks p.i., along with control sera from non-infected pigs at 4 (*n* = 4) and 8 (*n* = 3) weeks. HEV RNA quantification was performed by ORF3 RT-qPCR, as described previously [11]. Of note, the dynamics of HEV infection in pigs differ from those in humans. Persistent hepatitis E infection in pigs was defined as >8 weeks p.i., based on the literature [6,12].

### 2.2. Human Samples

Immunocompromised patients with positive HEV viremia followed up at Tours University Hospital, France, between 1 January 2011 and 31 December 2024 were considered for inclusion. For this study, we retrospectively included 9 immunocompromised patients with HEV infection (5 with chronic and 4 with resolutive infection). Immunosuppression was defined as a solid organ transplant, a hematopoietic stem cell transplant, or patients receiving chemotherapy for cancer diseases. Routine patient follow-up and HEV RNA quantification were performed as previously described [13]. For clarity, the date of diagnosis of HEV infection was considered day 0 of HEV infection. Inclusion criteria were patient follow-up until HEV cure (undetectable HEV RNA in sera and feces) and the availability of frozen serum samples for the miRNA study. HEV RNA quantification was performed using Realstar HEV RT-PCR kit 1.0/2.0, Altona Diagnostics, France. Patients were divided into two groups: resolving infection (clearance of HEV RNA in sera and feces before 3 months post diagnosis) and chronic infection (≥3 months of infection) [14]. Likewise, paired samples from longitudinal patient follow-up were identified as early (<3 months), late (≥3 months), or control (after HEV clearance in sera and feces). This study was approved by the ethics committee of Tours University (No. 2019 071). Patients involved in the study were informed before inclusion (see Informed Consent Statement).

### 2.3. Library Preparation and Small RNA Sequencing

For small RNA sequencing, total RNA was isolated from 200 μL using the miRNeasy Serum/Plasma Kit (QIAGEN, Heidelberg, Germany) according to the manufacturer’s instructions with an elution volume of 14 μL. For sequencing, control samples from healthy (*n* = 3) and immunocompromised (*n* = 3) pigs and samples from immunocompromised and HEV-infected pigs (*n* = 10) at 10 weeks p.i. were used. Small RNA sequencing and bioinformatic analysis were performed by QIAGEN Service (Heidelberg, Germany). Quality control criteria were max group mean Reads per kilo base per million mapped reads (RPKM) > 100 and false discovery rate (FDR)-adjusted *p*-value < 0.05.

### 2.4. qPCR Quantification of microRNAs

To explore miRNA response associated with HEV infection in immunocompromised pigs, 23 miRNAs of interest (Supplementary Table S1) were selected based on their fold change in RNAseq (≤−2.15 or ≥2.68; *n* = 21) or their relevance in the literature (ssc-miR-30a-5p and ssc-miR-192-5p) [1,2]. Among these, 17 miRNAs were successfully amplified. Five miRNAs with stable expression in RNAseq (ssc-miR-30d, ssc-miR-7134-3p, ssc-miR-421-3p, ssc-miR-151-5p, and ssc-miR-425-5p) were included for potential use as controls for normalization. Among these, two miRNAs (ssc-miR-30d and ssc-miR-425-5p) initially considered as controls showed variation over time and were reclassified as miRNAs of interest (*n* = 19). Following qPCR, normalization was performed using the global mean Ct of all expressed miRNAs instead of using these five miRNAs, because of variability in the expression levels of these five candidate control miRNAs. Four spike-in controls (UniSp2, UniSp3, UniSp4, and UniSp6) were also amplified for quality control.

To explore miRNA response associated with HEV infection in immunocompromised patients, 28 miRNAs of interest were quantified by qPCR (Supplementary Table S4). These miRNAs were selected because of their association with hepatic diseases [1,2,4,15,16,17,18,19,20,21] and/or for their relevant RNAseq profile in the pig model (miR-122-5p, miR-30a-5p, miR-27a, miR-99a-5p, miR-590-5p, miR-181a-5p, miR-451a, miR-23a-3p, and let-7i-5p) or previously described as controls. Normalization was performed using global mean Ct as described above. Briefly, total RNA enriched for small RNAs was extracted from 200 µL of serum using the miRNeasy Serum/Plasma Advanced Kit (QIAGEN), with spike-in controls (UniSp2, UniSp4, UniSp5) added to Buffer RPL to monitor extraction efficiency. After lysis, clarification, isopropanol precipitation, and purification on RNeasy UCP MinElute columns, RNA was eluted in 20 µL of RNase-free water. For qPCR validation, circulating miRNAs were reverse-transcribed using the miRCURY LNA RT Kit (QIAGEN) with 1 µL of RNA per 10 µL of reaction and UniSp6 as the RT control, using an incubation of 42 °C for 60 min followed by enzyme inactivation at 95 °C for 5 min. miRNA expression was quantified with the miRCURY LNA SYBR Green PCR Kit (QIAGEN) on custom 384-well panels, using cDNA diluted 1:40 in 10 µL reactions and run on a Roche LightCycler 480 II (95 °C 2 min; 45 cycles of 95 °C 10 s and 56 °C 60 s). Non-template controls and inter-plate calibration (UniSp3) were included, where spike-ins monitored extraction/RT performance, and hemolysis was assessed using the ΔCq (miR-23a-3p miR-451a < 7) metric.

### 2.5. Statistical Analysis

Raw Ct values were processed using the Qiagen GeneGlobe Data Analysis platform, applying a Ct ≤ 37 threshold to ensure inclusion of only robust amplification signals. Normalization was performed using the global mean Ct of all expressed miRNAs, which provides a stable reference for circulating miRNA datasets. ΔCt values were subsequently calculated, and fold changes were derived using the 2^−ΔΔCt^ method. The distribution of normalized ΔCt values was assessed using the Shapiro–Wilk normality test before downstream statistical analyses. In humans, longitudinal serum samples collected from the same patients during the acute, chronic, and cured (control) phases allowed paired analyses of ΔCt and fold changes (FCs). Statistical comparisons between these paired clinical groups were performed using two-tailed Student’s *t*-tests for normally distributed ΔCt values, with *p* < 0.05 considered significant. In pigs, serum samples from 0, 4, 8, 10, and 11 weeks p.i. originated from the same infected animals, while control samples corresponded to independent animals sampled at 4 and 8 weeks p.i.; therefore, analyses were unpaired. Statistical comparisons across groups (0, 4, 8, 10, 11 weeks p.i. and controls) were performed using two-tailed Student’s *t*-tests for normally distributed ΔCt values, with *p* < 0.05 considered significant. Scatter plots with SEM were generated using GraphPad Prism 10.6.1.

Correlation analyses between serum viral load (log_10_ IU/mL) and individual miRNA expression levels (ΔCt) were performed separately for each infection time point (4, 10, and 11 weeks p.i.) using Pearson correlation, as the variables had a normal distribution (Shapiro–Wilk normality). To evaluate whether the strength or direction of these correlations differed across infection stages, correlation coefficients (Pearson’s r) obtained at each week were compared pairwise (10 vs. 11 weeks p.i., 10 vs. 4 weeks p.i., and 11 vs. 4 weeks p.i.) using Fisher’s r-to-z transformation. Statistical significance was set at *p* < 0.05, and trends were considered for *p* < 0.20 due to the small sample sizes at each time point.

## 3. Results

### 3.1. A miRNA Signature Is Associated with Persistent HEV Infection in Pigs

**Figure 1:**
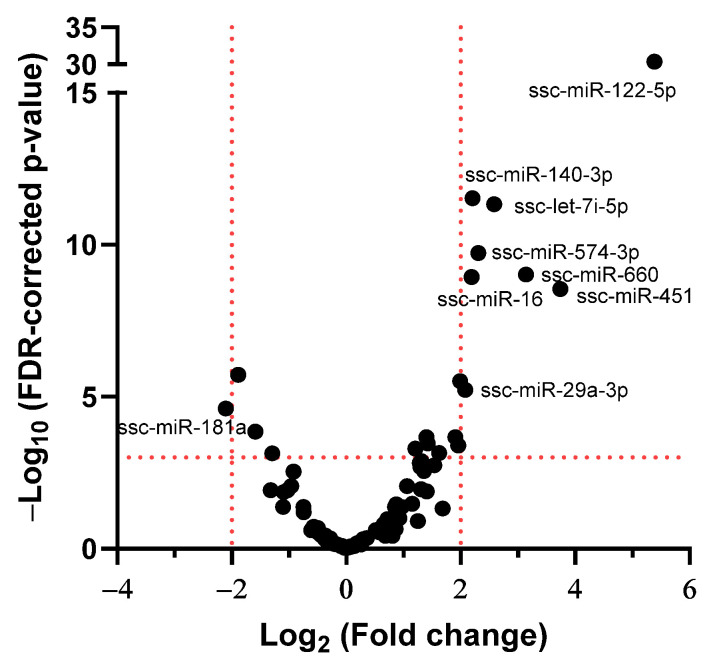
MicroRNA expression during persistent HEV infection (10 weeks p.i.) in immunocompromised pigs, using non-HEV-infected immunocompromised pigs as controls. Volcano plot derived from small RNA-sequencing data. Red dashed lines correspond to *p* < 0.001 and FC < −4 or >4.

We aimed to identify the miRNA signature associated with persistent HEV infection in immunocompromised pigs, followed up to 11 weeks p.i. (Supplementary Figure S1). To this aim, we performed small RNA sequencing (RNAseq, QIAseq miRNA, QIAGEN, and NextSeq, Illumina, San Diego, CA, USA) and qPCR on serum samples (collected 10 weeks p.i.) in HEV-infected immunocompromised pigs compared to HEV non-infected immunosuppressed pigs. After quality control analysis, 85 out of 108 detectable miRNAs were selected for RNAseq analysis (Supplementary Table S1). Among these, eight miRNAs were upregulated (FC > 4, *p* < 0.001) and one miRNA was downregulated (FC < −4, *p* < 0.001) in pigs with persistent HEV infection (Figure 1). These corresponded to ssc-miR-122-5p (FC = 41.86), ssc-miR-451a (FC = 13.38), ssc-miR-660 (FC = 8.83), let-7i-5p (FC = 6.02), ssc-miR-574-3p (FC = 4.96), ssc-miR-140-3p (FC = 4.62), ssc-miR-16 (FC = 4.56), ssc-miR-29a-3p (FC = 4.22), and ssc-miR-181a (FC = −4.28).

### 3.2. Time Course of miRNA Response During Persistent HEV Infection in Pigs

**Figure 2:**
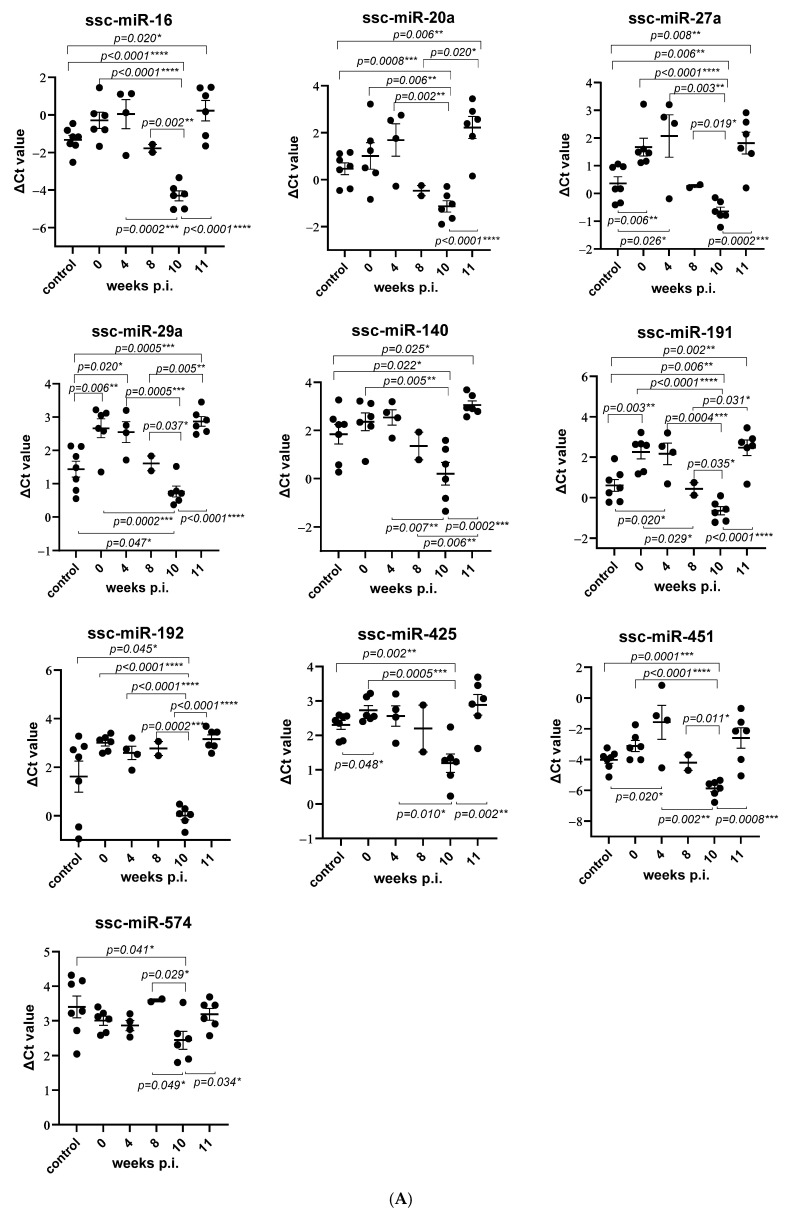

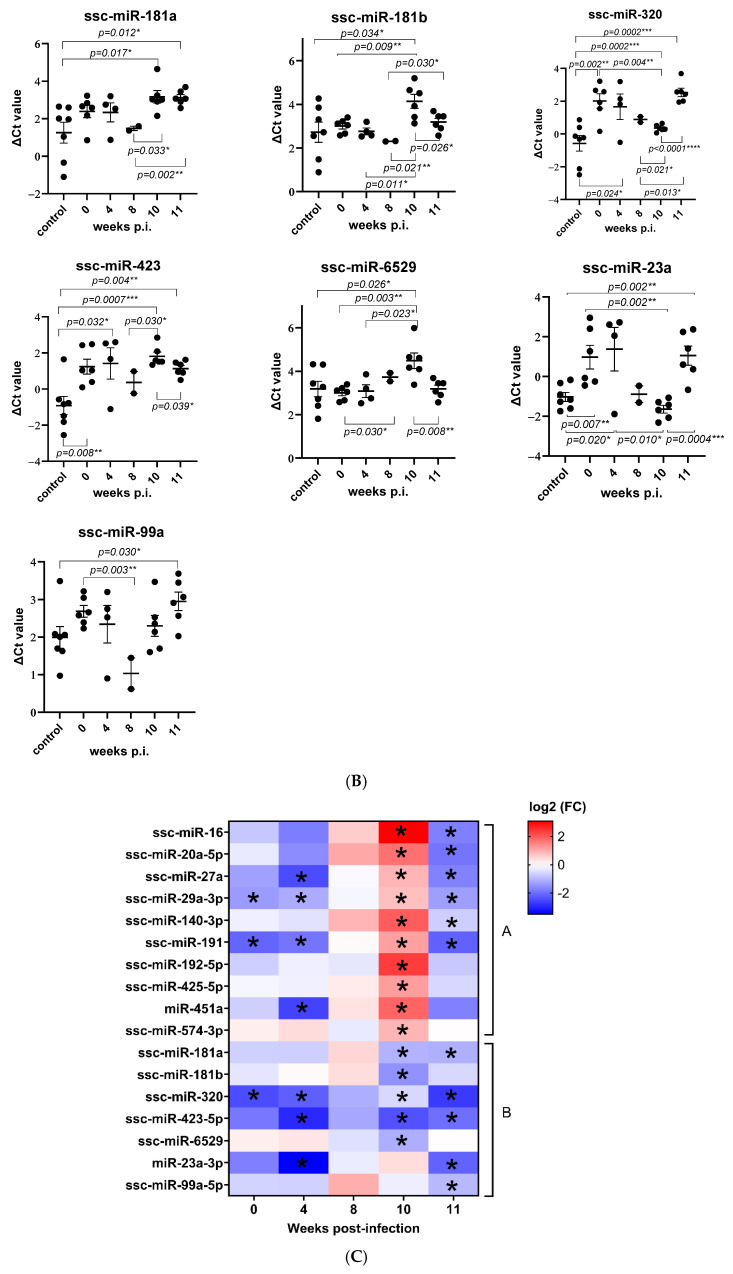
Quantification of microRNA expression at 0, 4, 8, 10, and 11 weeks post-infection in HEV-infected immunosuppressed pigs compared to control pigs (immunosuppressed non-infected). (**A**,**B**). ΔCt-based analysis illustrates miRNA expression compared to control miRNAs in the same sample. Noting that ΔCt values are inversely proportional to fold change in expression levels. (**A**) MiRNAs upregulated 10 weeks post-infection (weeks p.i.). (**B**) MiRNAs downregulated 10 or 11 weeks p.i. (**C**) Heatmap showing log2 fold change (FC) in serum miRNA expression levels in immunosuppressed HEV-infected pigs compared to control pigs. Group A corresponds to miRNAs upregulated at 10 weeks p.i. Group B corresponds to other miRNAs downregulated at 10 or 11 weeks p.i. Asterisks indicate significant variations (*p* * < 0.05, *p* ** < 0.01, *p* *** < 0.001, *p* **** < 0.0001) compared to controls.

To better understand the miRNA response during persistent HEV infection, we quantified the miRNA expression levels at 0, 4, 8, 10, and 11 weeks p.i. in HEV-infected immunocompromised pigs. A total of 19 miRNAs of interest were successfully amplified, among which 17 showed significant variations (*p* < 0.05) compared to control pigs (Figure 2).

A first group of 10 miRNAs was significantly upregulated at 10 weeks p.i. compared to controls (Figure 2A,C). These corresponded to ssc-miR-16, ssc-miR-29a-3p, ssc-miR-140-3p, ssc-miR-451a, and ssc-miR-574-3p previously identified by RNAseq, along with ssc-miR-20a-5p, ssc-miR-27a, ssc-miR-191, ssc-miR-192-5p, and ssc-miR-425-5p. Interestingly, the expression levels of these 10 miRNAs significantly decreased between 10 and 11 weeks p.i (Figure 2). At 11 weeks p.i., the expression level of six of these miRNAs was significantly lower than that of controls. A second group of 7 miRNAs was downregulated at 10 and/or 11 weeks p.i. These corresponded to ssc-miR-181a, ssc-miR-181b, ssc-miR-320, ssc-miR-423-5p, and ssc-miR-6529 (Figure 2B,C). Of note, ssc-miR-122-5p upregulation could not be confirmed due to amplification issues. Also, no significant differences were observed between 0 and 4 weeks p.i., indicating that modulation of circulating miRNAs does not occur immediately after infection in this pig model, and divergences appear from 8 to 10 weeks p.i. Overall, these longitudinal data demonstrate that circulating miRNAs undergo pronounced and synchronized remodeling during HEV infection in pigs. Overall, these longitudinal data demonstrate a synchronized regulation of miRNA expression during HEV infection in pigs, in particular at 10 and 11 weeks p.i.

### 3.3. MiRNAs Associated with the Control of HEV Viremia During Persistent HEV Infection in Pigs

**Figure 3:**
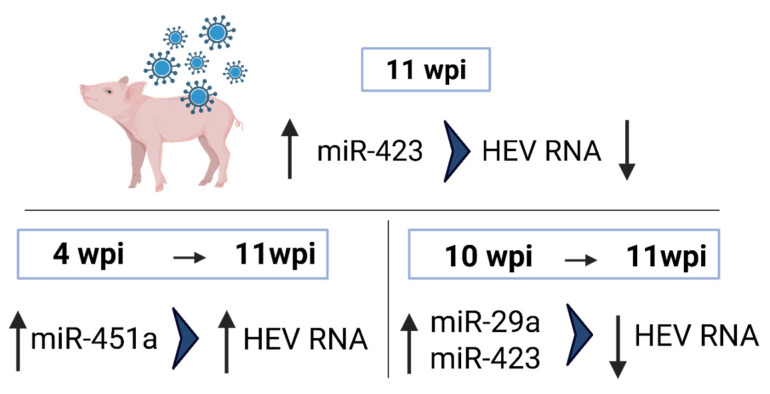
MiRNAs are associated with the control of HEV viremia during persistent HEV infection. In the upper part, arrows indicate the direct or inverse association between serum miRNA expression and serum HEV RNA at 11 weeks p.i. In the lower part, they indicate the association between variation in serum miRNA levels and variation in HEV RNA levels during follow-up.

In our pig model, HEV viremia (i.e., RNA in sera) increased between 1 and 3 weeks p.i. (*p* = 0.012), stabilized at 5 log_10_ IU/mL between 3 and 10 weeks p.i., then decreased between 10 weeks p.i. and the end of the study at 11 weeks p.i. (4 log_10_ IU/mL, *p* = 0.008) (Supplementary Figure S1). In this context, we retrospectively searched for an association between miRNA expression and HEV viremia at relevant timepoints (0, 4, 8, 10, and 11 weeks p.i.). Correlation between viremia and weekly miRNA levels was assessed using Pearson’s correlation (Supplementary Table S2). Because dCt values are inversely proportional to miRNA expression, positive r values indicate an inverse correlation between miRNA expression and HEV viremia. At 11 weeks p.i., higher expression of ssc-miR-423-5p (i.e., lower ∆Ct) was associated with reduced HEV viremia (r = 0.914; *p* = 0.011) (Figure 3).

To better understand the relationship between miRNAs and HEV viremia, we explored how the correlation between viremia and miRNA expression differs between early and late stages of infection, using Fisher’s z transformation (Supplementary Table S2). In this context, a z-value < 0 (with a *p*-value < 0.05) means that r_early_ < r_late_. In this example, “r” increases over time, suggesting a trend towards an inverse correlation between miRNA expression and HEV viremia. This was observed for ssc-miR-423-5p between 10 and 11 weeks p.i. (r_10 weeks p.i._ = −0.506 to r_11 weeks p.i._ = 0.914, *p* = 0.005), suggesting an evolution toward an inverse correlation with HEV viremia, as described above. Ssc-miR-29a-3p exhibited a similar switch at the same time (from r = −0.618 to r = 0.687; *p* = 0.028). In contrast, ssc-miR-451a expression evolved towards a positive association with HEV viremia between 4 and 11 weeks p.i. (from r = 0.785 to r = −0.685, *p* = 0.05). Collectively, these results indicate that ssc-miR-423-5p and ssc-miR-29a-3p have a negative association with HEV viremia between 10 and 11 weeks p.i., while ssc-miR-451a has a positive association with HEV viremia between 4 and 11 weeks p.i. (Figure 3).

### 3.4. MiRNAs Are Associated with Resolving HEV Infection in Immunocompromised Patients

For this study, we retrospectively included nine patients: Five in the chronic group and four in the resolving group. Their clinical and biological features are presented in Table 1 and Supplementary Table S3. A total of 22 serum samples were available for these patients. They corresponded to at least one early, one late (for the chronic group), and one control serum sample for each patient (except for patient C16, who had no early sample) (Supplementary Table S3). Based on miRNA sequencing in pigs and data from the literature, we quantified the expression levels of 20 relevant miRNAs in these samples (a complete list of miRNAs is in Supplementary Table S4). Volcano plot analysis revealed a miRNA signature specific of immunocompromised patients with resolving HEV infection, characterized by an upregulation (FC > 2, *p* < 0.05) of hsa-miR-99a-5p, hsa-miR-122-5p, hsa-miR-125b-5p, and hsa-miR-194-5p, with a downregulation of miR-106a-5p (FC < −2, *p* < 0.05) during the first three months of HEV infection, compared to paired control samples collected after HEV cure in the same patients. Of note, during the first three months of infection, a downregulation of miR-221-3p (FC < −2, *p* < 0.05) was observed in both resolving and chronic groups.

**Figure 4:**
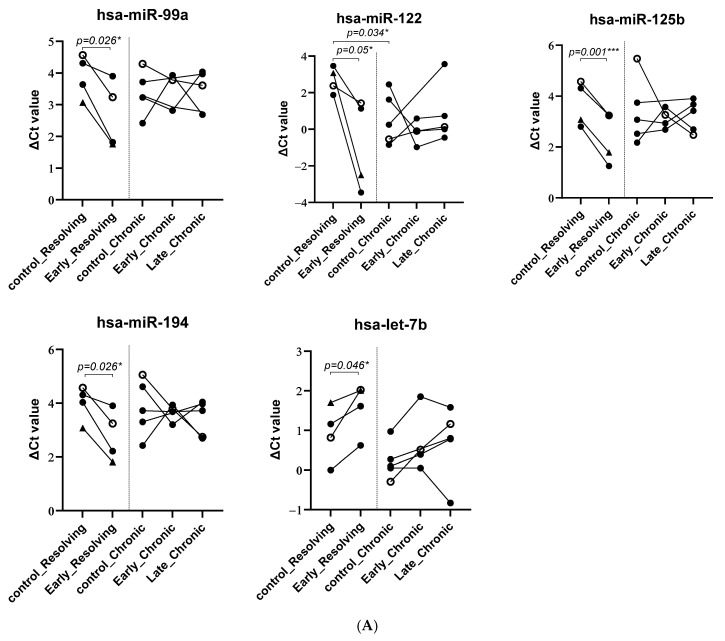

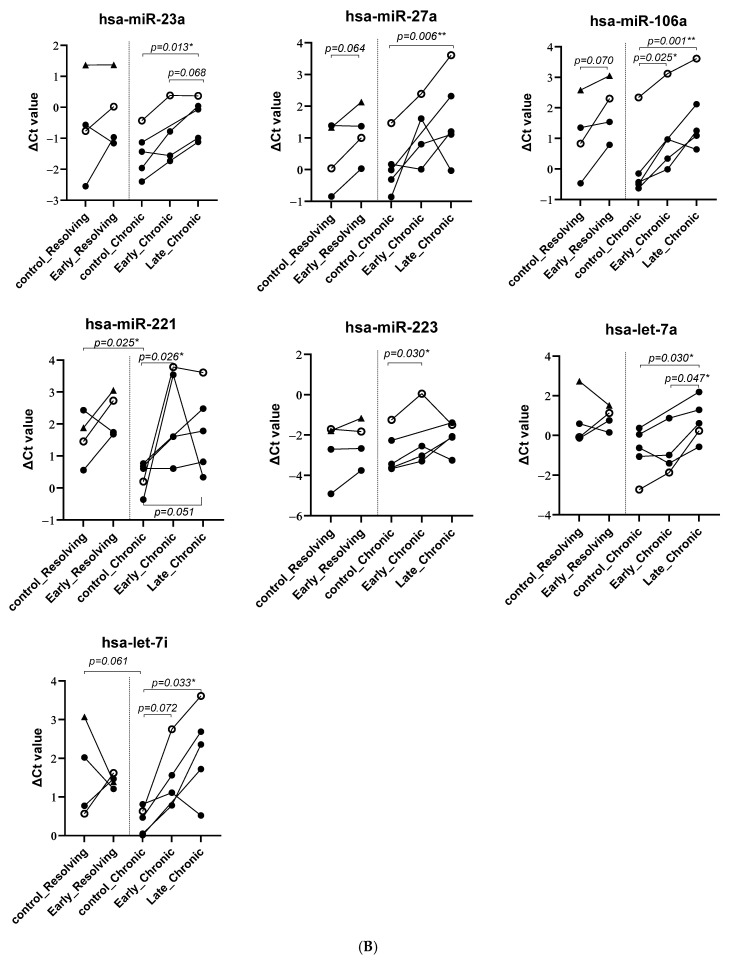
Individual follow-up of microRNA expression in immunocompromised patients with HEV infection. (**A**,**B**) ΔCt-based analysis illustrating (**A**) the miRNA signature associated with immunocompromised patients experiencing resolving HEV infection and (**B**) the miRNA signature associated with chronic HEV infection. Full circles, kidney transplant; empty circles, heart transplant; triangles, multiple myeloma. Asterisks indicate significant variations (p * < 0.05, *p* ** < 0.01, *p* *** < 0.001) compared to controls.

Analysis of ΔCt values confirmed that hsa-miR-99a-5p (*p* = 0.026), hsa-miR-122-5p (*p* = 0.05), hsa-miR-125b-5p (*p* = 0.001), and hsa-miR-194-5p (*p* = 0.026) were significantly upregulated in immunocompromised patients experiencing spontaneously resolving HEV infection (Figure 4A), and also identified a downregulation of hsa-let-7b-5p (*p* = 0.046) in these patients (Figure 4A). In contrast, another set of miRNAs (miR-23a-3p, miR-27a, miR-106a-5p, miR-221-3p, and miR-223-3p) was downregulated only in patients with chronic HEV infection (Figure 4B). Of note, expression of hsa-miR-122-5p (*p* = 0.034) and hsa-miR-221-3p (*p* = 0.025) in controls differed between the resolving and chronic group, highlighting the relevance of considering each patient as its own control (Figure 4B). These inter-group differences could predate HEV infection (no sample available) or could suggest a long-lasting impact of HEV infection on miRNA responses. We aimed to determine if these differences in miRNA expression were specific to the transplant type. We confirmed an association among miR-23a, miR-27a, miR-106a, and chronic HEV infection (*p* < 0.05) in kidney transplant recipients. Other associations were not confirmed, and other types of immunosuppression were not explored due to the small sample size.

Altogether, these results revealed two miRNA signatures when considering each patient after HEV cure as its own control. First, miR99a, miR-122-5p, miR-125b-5p, miR-194-5p, and let-7b-5p were associated with the resolution of the HEV infection in immunocompromised patients. Second, miR-23a-3p, miR-27a, miR-106a-5p, and miR-221-3p were associated with chronic HEV infection.

### 3.5. MiRNAs Associated with HEV Viremia in the Late Stage of Chronic HEV Infection in Immunocompromised Patients

**Figure 5:**
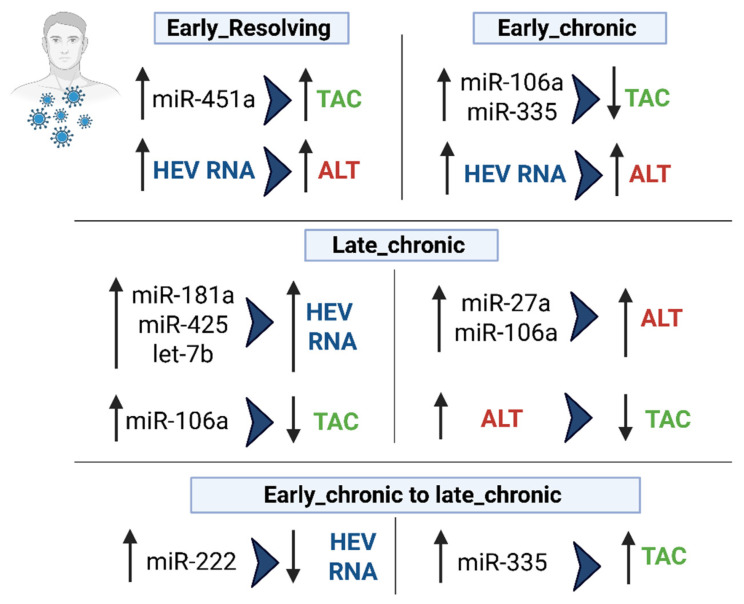
Correlation between miRNA expression levels, HEV RNA quantification, Alanine transferase levels (ALT), and residual concentration of tacrolimus (TAC) in patients with resolving HEV infection (Early_resolving), or chronic HEV infection, either during the early (<3 months, early_chronic) or late stages of infection (≥3 months). Arrows indicate the variation (up ↑ or down ↓) of parameters analyzed (miRNAs, TAC, ALT, HEV RNA).

Correlation analysis between HEV viremia and miRNA ΔCt values showed distinct patterns by patient group and sample time. During the late stage of chronic HEV infection, several miRNAs were positively correlated with HEV viremia: hsa-miR-181a-5p (r = −0.965, *p* = 0.008), hsa-miR-425-5p (r = −0.964, *p* = 0.008), and hsa-let-7b-5p (r = −0.935, *p* = 0.020) (Supplementary Table S5, Figure 5). In addition, comparison of Pearson’s correlation coefficients using Fisher’s r-to-z transformation indicated that hsa-miR-222-3p evolved towards an inverse correlation with HEV viremia between early and late samples in the chronic group (r_early_ = −0.926 to r_late_ = 0.415, *p* = 0.046) (Supplementary Table S5, Figure 5).

### 3.6. Stage-Dependent Associations Between Circulating microRNAs, Liver Injury, HEV Viremia, and Immunosuppression in Patients

ALT levels, reflecting liver injury, were positively correlated with expression levels of miR-27a-3p (r = −0.884, *p* = 0.046) and miR-106a-5p (r = −0.920, *p* = 0.027) in the late stages of chronic HEV infection (Figure 5 and Supplementary Table S6). Tacrolimus trough concentrations were negatively correlated with expression levels of miR-335-5p during the early stages of HEV infection (r = 0.994, *p* = 0.006), and with expression levels of miR-106a-5p during late stages of chronic HEV infection (r = 0.934, *p* = 0.020). Tacrolimus trough concentrations were not significantly different between stages of HEV infection and between resolving and chronic patients (Supplementary Table S3 and Supplementary Figure S2). This suggests that tacrolimus trough concentration was not a confounding factor in the association described above between mir-106a-5p expression and chronic HEV infection. Tacrolimus trough concentrations were positively correlated with miR-451a expression in patients experiencing resolving HEV infection (r = −0.951, *p* = 0.049) (Figure 5 and Supplementary Table S7). Finally, we observed a positive correlation between HEV viremia and ALT during the first three months of infection, both in the resolving (r = 0.987, *p* = 0.013) and chronic groups (r = 0.964, *p* = 0.036). We also observed a negative correlation between ALT and tacrolimus trough concentrations in the context of chronic HEV infection (r = −0.894, *p* = 0.041) (Figure 5 and Supplementary Table S8).

### 3.7. Comparative Analysis of miRNA Profiles Between HEV-Infected Immunocompromised Pigs and Patients

**Figure 6:**
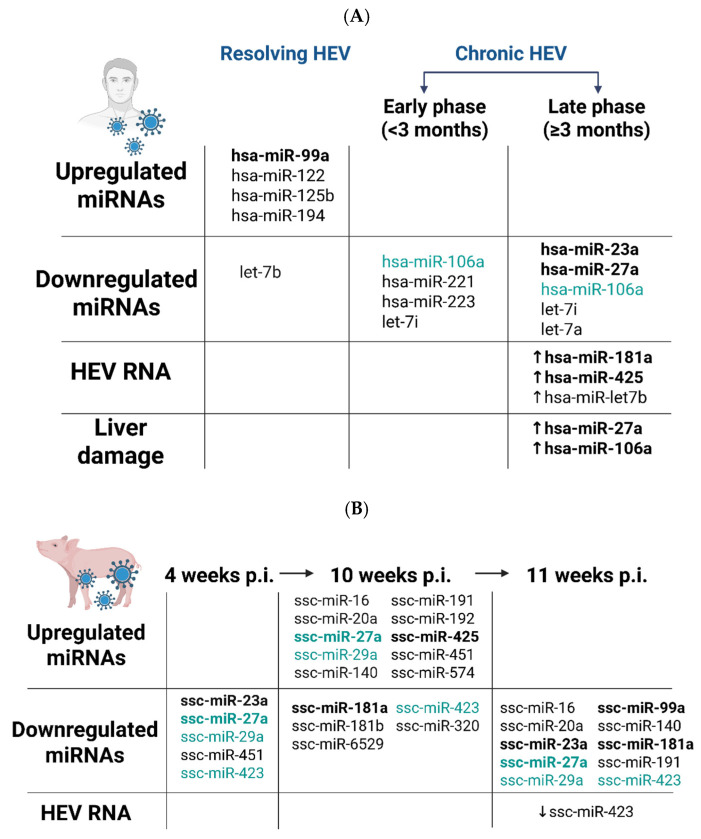
miRNA expression level during HEV infection. (**A**) miRNA dysregulation in immunocompromised patients during resolving and chronic HEV infection. (**B**) miRNA dysregulation in immunocompromised pig model during 4 w.p.i., 10 w.p.i., and 11 w.p.i. Only miRNAs with significant variations compared to controls (*p* < 0.05, data above) are shown. In turquoise, miRNAs with variations in more than one group. In bold, miRNAs with variations both in pigs and humans. Arrows indicate up- (↑) or down- (↓) regulation of miRNAs.

Five miRNAs demonstrated significant variations in their expression levels in both immunocompromised pigs and humans. These were miR-23a-3p, miR-27a, miR-99a-5p, miR-181a, and miR-425-5p (Figure 6). It is difficult to compare results between pigs and humans, as the genetic host background and pathology of hepatitis E are known to differ between these hosts. In addition, the lack of pre-infection samples from patients and of paired control samples (cure) in pigs further prevents direct comparison of results. Yet, our results suggest similar miRNAs are associated with hepatitis E in immunocompromised hosts. In particular, miR-23a-3p and 27a were downregulated during the late stages of infection, both in pigs (11 weeks p.i.) and in chronically infected patients with HEV (≥3 months). These two miRNAs could play a role in the late stages of HEV infection in immunocompromised hosts. MiR-99a-5p was upregulated in patients with resolving HEV infection (*p* = 0.026). In contrast, it was downregulated during the late stages of infection in pigs (11 weeks p.i.). MiR-99a-5p seems to be involved at different timepoints of the infection depending on the host. Last, miR-181a and miR-425-5p were positively correlated with viremia during the late stage of chronic HEV infection in humans (miR-181a, *p* = 0.008; miR-425-5p, *p* = 0.008). There was a trend towards such an association in pigs. Indeed, miR-425-5p was upregulated 10 weeks p.i, when HEV viremia was high (5 log IU/mL), while miR-181a was downregulated 11 weeks p.i., when HEV viremia was lower. Together, these findings highlight some similarities in miRNA responses during HEV infection in humans and pigs. This supports the translational relevance of the porcine model in understanding the pathology of HEV infection in humans.

## 4. Discussion

Our study revealed two miRNA signatures that allow for the distinction between immunocompromised patients experiencing resolving HEV infection and patients with chronic HEV infection. Indeed, resolutive HEV infection was specifically associated with upregulation of miR-99a-5p, miR-122-5p, miR-125b-5p, and miR-194-5p, and with downregulation of let-7b-5p during the first three months of HEV infection. In contrast, late stages of chronic HEV infection (≥3 months) were associated with a downregulation of miR-23a-3p, miR-27a, miR-106a-5p, miR-221-3p, and miR-223-3p. We also revealed similarities in miRNA responses between immunocompromised patients and a pig model. In particular, miR-23a-3p and miR-27a were downregulated during the late stages of HEV infection in both patients and pigs, suggesting that they might be relevant for the persistence of HEV infection in both hosts. Other miRNAs (miR-99a-5p, miR-122-5p, miR-181a, and miR-425-5p) showed significant variations in both hosts but at different timepoints. Mechanistic studies are required to determine the role of these miRNAs in the pathogenesis of HEV infection.

Previous studies revealed specific miRNA signatures in patients with acute and chronic HEV infection [1,2]. Briefly, Costafreda et al. (2023) reported that acute and chronic HEV genotype 3 infections were associated with upregulation of miR-30a, miR-122, miR-194, and miR-885, while miR-27a, miR-221, and miR-223 were downregulated, compared to immunocompetent controls [1]. They also revealed that upregulation of miR-885 allowed to discriminate chronic HEV infection from acute HEV infection [1]. Harms et al. (2020), reported that miR-99a-5p, miR-122-5p, and miR-125b-5p were elevated in both acute and chronic HEV patients compared to immunocompromised controls [2]. MiRNA response in patients with chronic HEV infection was characterized by higher miR-192-5p expression than in controls and a lower expression of miR-99a and miR-125b than in patients with acute hepatitis E [2]. In line with these studies, we confirmed that HEV infection in immunocompromised patients is associated with upregulation of miR-99a, miR-125b [2], miR-122-5p [1,2], and miR-194-5p [1], and downregulation of miR-27a [1], miR-221, and miR-223 [2]. Importantly, our study revealed that these changes occur in a timely manner and differ between resolving and chronic HEV infection.

Previous studies have revealed specific miRNA responses associated with chronic HEV infection in immunocompromised patients, but the early miRNA response (<3 months) in these patients remains unexplored. Yet, these early phases of HEV infection in immunocompromised patients are crucial to understand the evolution towards resolutive or chronic HEV infection. On this basis, we chose to explore miRNA responses during HEV infection by longitudinal follow-up of immunocompromised patients who either resolved or developed chronic HEV infection. This approach allowed us to reveal upregulation of miR-99a-5p, miR-125b-5p, miR-122-5p, and miR-194-5p during the acute phase of HEV infection in immunocompromised patients evolving towards resolutive infection, which was not observed in patients evolving towards chronic HEV infection. Regarding miR-99a-5p and miR-125b-5p, our results relate to those of Harms et al., who described higher levels of these miRNAs during acute HEV infection in immunocompetent patients than during chronic HEV infection [2]. The upregulation of miR-99a-5p and miR-125b-5p appears to be a common pattern between acute HEV infection in immunocompetent patients and resolutive HEV infection in immunocompromised patients. Thus, we hypothesize that miR-99a-5p and miR-125b-5p may serve as early markers of a resolutive HEV infection. If confirmed, this could enable discrimination between immunocompromised patients at low and high risk of progression to chronicity, allowing targeted and early intervention. We also observed upregulation of miR-122-5p and miR-194-5p in immunocompromised patients progressing toward resolutive HEV infection. MiR-122-5p is the most abundant miRNA in the liver. It has been associated with increased HEV replication in vitro [4] and liver damage (elevated ALT) during chronic HEV infection [2]. Both miR-122-5p and miR-194-5p have been associated with symptomatic acute HEV-3 infection in immunocompetent hosts [1]. Last, miR-99a is known to restrict HCV replication [22] and miR-125b-5p has been associated with the regulation of the IFN pathways [19]. Functional assays would be relevant to determine whether these miRNAs are associated with the immune response against HEV, viral replication, symptomatic HEV infections, and an evolution towards a resolutive HEV infection. Unlike previous studies, we did not observe any association between miR-122-5p [1,2] or miR-194-5p [1] and chronic HEV infection. This could be due to different study designs and definitions of controls.

We observed an association between late stages of chronic HEV infection (≥3 months) and a downregulation of miR-23a-3p, miR-27a, miR-106a-5p, miR-221-3p, and miR-223-3p. Among these miRNAs, miR-221-3p has previously been associated with the modulation of the immune response in the liver and liver disease [23,24]. Its expression can be downregulated by the HEV ORF3 protein in vitro [25]. Our results confirm the relevance of miR-221-3p in the context of HEV infection and support further functional studies to decipher the role of miR-221-3p and ORF3 in the pathogenesis of Hepatitis E. Other miRNAs downregulated during chronic HEV infection may also contribute to pathogenesis, based on their role in other liver diseases. In particular, miR-27a controls hepatitis C virus replication [26], while miR-106a is involved in cell-cycle regulation and the pathogenesis of several cancers [27], and miR-223 is described as an important regulator of liver metabolism and innate immune responses [28]. Overall, these results prompt further in vitro studies to determine the impact of a downregulation of miR-23a-3p, miR-27a, miR-106a-5p, miR-221-3p, and miR-223-3p on viral replication, liver disease, and immune response.

During persistent HEV infection in pigs (10 weeks p.i.), several circulating miRNAs were differentially expressed. MiR-29a-3p levels were increased, a pattern previously associated with a protective role in an animal model of non-alcoholic steatohepatitis (NASH) [29]. MiR-181a levels were decreased, as observed in patients with a good prognosis in the context of acute liver failure [30]. Overall, the expression profiles of these two miRNAs were known to be associated with protective factors in liver disease. Our results push for mechanistic studies to explore the interplay between these miRNAs and liver pathology. MiR-20a-5p expression was increased, a pattern observed in patients progressing from acute to chronic hepatitis C virus infection [31]. We also observed upregulation of miR-140, which has been reported to promote efficient HEV replication [32], as well as increased expression of miR-192, previously described as elevated in patients with chronic HEV infection [2]. Our observations push for further studies to explore the roles of these miRNAs in HEV replication. At 11 weeks p.i., additional alterations were detected. In particular, miR-27a expression was decreased, consistent with reports in both acute and chronic HEV-infected patients [1]. Moreover, miR-16 was downregulated, a pattern previously described in patients with hepatocellular carcinoma and chronic liver diseases [33].

We acknowledge that this study has limitations, including a relatively small sample size, which could limit statistical power and the generalizability of the results. Furthermore, potential confounding factors related to host characteristics, ribavirin exposure, and clinical heterogeneity, along with the lack of mechanistic validation experiments, limit understanding of the roles of microRNAs identified during HEV infection.

## 5. Conclusions

In summary, our findings demonstrate that a subset of miRNAs is associated with patient outcome (resolving or chronic infection) and stage of infection (early or late) in immunocompromised patients experiencing HEV infection. This observational clinical study argues for further in vitro and in vivo characterization of these miRNAs to better understand the pathogenesis of HEV infection. If our results are confirmed in larger cohorts, circulating miRNAs could be used as stage-specific biomarkers for HEV infection. In immunocompromised patients, where early identification of non-resolvers is critical, miRNA profiling could enable risk stratification and personalized therapeutic decisions (such as who may benefit from preemptive antiviral therapy). As no single clinical parameter currently suffices to predict HEV chronicity or relapse, integrating miRNA biomarkers into routine patient follow-up could improve the monitoring and risk stratification of HEV infection. Our translational pig model reinforces these concepts by demonstrating a partial overlap between miRNA signatures of HEV infection in immunocompromised humans and pigs. This provides confidence that these biomarkers are biologically relevant across species and that immunosuppressed swine is a faithful model of HEV infection. This pig model can be further exploited to dissect the cause-and-effect relationships between miRNA changes and viral control, and to test interventions (for example, miRNA mimics or inhibitors) in a preclinical setting. In conclusion, our study contributed to the growing body of evidence that circulating miRNAs are dynamic sentinels of HEV infection. Mechanistic studies are now required to determine the role of these miRNAs in the pathogenesis of HEV infection in both hosts. Further clinical evaluation of relevant miRNAs would also help assess their potential as new biomarkers or antiviral targets, ultimately improving outcomes for vulnerable patients facing this emerging viral disease.

## Figures and Tables

**Table 1:** Clinical features of HEV infection in immunocompromised patients.

Patient	Group	Gender	Age	Immunosuppression	Delay Between Start of Immunosuppression and HEV Infection (Months)	Delay Before Ribavirin Start (Months)	Duration of Ribavirin Treatment (Months)	Duration of HEV RNA Detection in Sera (Months)	Duration of HEV RNA Detection in Feces (Months)
C10	Chronic	F	63	Kidney transplant	68	6.0	3.0	6.3	6.3
C16	Chronic	M	54	Kidney transplant	334	1.7	24.3	24.8	24.8
C18	Chronic	F	53	Kidney transplant	29	3.7	2.5	5.6	4.6
C9	Chronic	F	0	Heart transplant	1368	0.6	17.6	38.2	31.7
P20	Chronic	M	65	Kidney transplant	108	5.0	4.0	4.0	5.2
P3	Resolving	M	0	Kidney transplant	80	2.3	4.4	2.3	1.2
P30	Resolving	M	0	Kidney transplant	65	0.7	8.5	1.4	0.7
P50	Resolving	F	0	Multiple myeloma	23	0.6	8.3	1.6	0.9
P53	Resolving	M	0	Heart transplant	73	3.2	4.5	2.6	N/A

## Data Availability

The original contributions presented in this study are included in the article and Supplementary Materials. Further inquiries can be directed to the corresponding authors.

## Supplementary Materials

The following supporting information can be downloaded at: https://www.mdpi.com/article/10.3390/biom16060799/s1. Figure S1. Quantification of HEV RNA in sera during 11 weeks of infection in immunocompromised pigs (modified from [6]). Circles on the *X*-axis (A) indicate the weeks from which the pig serum samples were extracted for this study. Figure S2. Individual follow-up of tacrolimus trough concentration in immunocompromised patients with HEV infection. Full circles, kidney transplant; empty circles, heart transplant. Table S1. Small RNAseq results in HEV-infected immunocompromised pigs and miRNAs considered for qPCR. Table S2. Correlation between HEV viremia (log UI/mL) in pigs and miRNA expression (ΔCt value). Table S3. Human sera samples selected for miRNA analysis. Table S4. Human miRNAs considered for qPCR. Table S5. Correlation between HEV viremia (log UI/mL) in patients and miRNA expression (ΔCt value). Table S6. Correlation between ALT and miRNA expression (ΔCt value) in patients. Table S7. Correlation between tacrolimus trough concentration and miRNA expression (ΔCt value) in patients. Table S8. Correlation between relevant clinical parameters in patients with HEV infection. References [1,2,5,19,20,30,32,34,35,36] are cited in the Supplementary Materials.

## References

[1] Costafreda M.I., Sauleda S., Riveiro-Barciela M., Rico A., Llorens-Revull M., Guix S., Pintó R.M., Bosch A., Rodríguez-Frías F., Rando A. (2023). Specific Plasma MicroRNA Signatures Underlying the Clinical Outcomes of Hepatitis E Virus Infection. Microbiol. Spectr..

[2] Harms D., Choi M., Allers K., Wang B., Pietsch H., Papp C.-P., Hanisch L., Kurreck J., Hofmann J., Bock C.-T. (2020). Specific circulating microRNAs during hepatitis E infection can serve as indicator for chronic hepatitis E. Sci. Rep..

[3] León-Janampa N., Marlet J. (2025). Differential miRNA signatures in Hepatitis E Virus Infection: Insights into acute, chronic, and pregnancy-related outcomes. Eur. J. Clin. Microbiol. Infect. Dis..

[4] Haldipur B., Bhukya P.L., Arankalle V., Lole K. (2018). Positive Regulation of Hepatitis E Virus Replication by MicroRNA-122. J. Virol..

[5] Patil R.N., Karpe Y.A. (2020). Uncovering the Roles of miR-214 in Hepatitis E Virus Replication. J. Mol. Biol..

[6] León-Janampa N., Caballero-Posadas I., Barc C., Darrouzain F., Moreau A., Guinoiseau T., Gatault P., Fleurot I., Riou M., Pinard A. (2023). A pig model of chronic hepatitis E displaying persistent viremia and a downregulation of innate immune responses in the liver. Hepatol. Commun..

[7] Gu X.-Q., Tang D., Wan P., Qin T., Yang T.-H., Wu J., Ji H., Liu J.-C., Xue F., Tang Y.-J. (2021). Multiple microRNAs regulate tacrolimus metabolism through CYP3A5. Pharmacol. Res..

[8] Hu J., Wang Z., Tan C.-J., Liao B.-Y., Zhang X., Xu M., Dai Z., Qiu S.-J., Huang X.-W., Sun J. (2013). Plasma microRNA, a potential biomarker for acute rejection after liver transplantation. Transplantation.

[9] Loga L., Dican L., Matei H.V., Mărunțelu I., Constantinescu I. (2022). Relevant biomarkers of kidney allograft rejection. J. Med. Life.

[10] Eckardt K.-U., Kasiske B.L., Zeier M.G. (2009). Special Issue: KDIGO Clinical Practice Guideline for the Care of Kidney Transplant Recipients. Am. J. Transplant..

[11] Abravanel F., Sandres-Saune K., Lhomme S., Dubois M., Mansuy J.-M., Izopet J. (2012). Genotype 3 Diversity and Quantification of Hepatitis E Virus RNA. J. Clin. Microbiol..

[12] Cao D., Cao Q.M., Subramaniam S., Yugo D.M., Heffron C.L., Rogers A.J., Kenney S.P., Tian D., Matzinger S.R., Overend C. (2017). Pig model mimicking chronic hepatitis E virus infection in immunocompromised patients to assess immune correlates during chronicity. Proc. Natl. Acad. Sci. USA.

[13] León-Janampa N., Boennec N., Le Tilly O., Ereh S., Herbet G., Moreau A., Gatault P., Longuet H., Barbet C., Büchler M. (2024). Relevance of Tacrolimus Trough Concentration and Hepatitis E virus Genetic Changes in Kidney Transplant Recipients with Chronic Hepatitis E. Kidney Int. Rep..

[14] Dalton H.R., Kamar N., Baylis S.A., Moradpour D., Wedemeyer H., Negro F. (2018). EASL Clinical Practice Guidelines on hepatitis E virus infection. J. Hepatol..

[15] Akamatsu S., Hayes C.N., Tsuge M., Miki D., Akiyama R., Abe H., Ochi H., Hiraga N., Imamura M., Takahashi S. (2015). Differences in serum microRNA profiles in hepatitis B and C virus infection. J. Infect..

[16] Barbu M.G., Condrat C.E., Thompson D.C., Bugnar O.L., Cretoiu D., Toader O.D., Suciu N., Voinea S.C. (2020). MicroRNA Involvement in Signaling Pathways During Viral Infection. Front. Cell Dev. Biol..

[17] Becker P.P., Rau M., Schmitt J., Malsch C., Hammer C., Bantel H., Müllhaupt B., Geier A. (2015). Performance of Serum microRNAs -122, -192 and -21 as Biomarkers in Patients with Non-Alcoholic Steatohepatitis. PLoS ONE.

[18] Giray B.G., Emekdas G., Tezcan S., Ulger M., Serin M.S., Sezgin O., Altintas E., Tiftik E.N. (2014). Profiles of serum microRNAs; miR-125b-5p and miR223-3p serve as novel biomarkers for HBV-positive hepatocellular carcinoma. Mol. Biol. Rep..

[19] Gupta S., Singh P., Tasneem A., Almatroudi A., Rahmani A.H., Dohare R., Parveen S. (2023). Integrative Multiomics and Regulatory Network Analyses Uncovers the Role of OAS3, TRAFD1, miR-222-3p, and miR-125b-5p in Hepatitis E Virus Infection. Genes.

[20] McGowan K., Simpson K.J., Petrik J. (2020). Expression Profiles of Exosomal MicroRNAs from HEV- and HCV-Infected Blood Donors and Patients: A Pilot Study. Viruses.

[21] Shrivastava S., Steele R., Ray R., Ray R.B. (2015). MicroRNAs: Role in Hepatitis C Virus pathogenesis. Genes Dis..

[22] Lee E.B., Sung P.S., Kim J.-H., Park D.J., Hur W., Yoon S.K. (2020). microRNA-99a Restricts Replication of Hepatitis C Virus by Targeting mTOR and de novo Lipogenesis. Viruses.

[23] Di Martino M.T., Arbitrio M., Caracciolo D., Cordua A., Cuomo O., Grillone K., Riillo C., Caridà G., Scionti F., Labanca C. (2022). miR-221/222 as biomarkers and targets for therapeutic intervention on cancer and other diseases: A systematic review. Mol. Ther. Nucleic Acids.

[24] Du H., Cui S., Li Y., Yang G., Wang P., Fikrig E., You F. (2018). MiR-221 negatively regulates innate anti-viral response. PLoS ONE.

[25] Cheng Y., Du L., Shi Q., Jiao H., Zhang X., Hao Y., Rong H., Zhang J., Jia X., Guo S. (2013). Identification of miR-221 and -222 as important regulators in genotype IV swine hepatitis E virus ORF3-expressing HEK 293 cells. Virus Genes.

[26] Shirasaki T., Honda M., Shimakami T., Horii R., Yamashita T., Sakai Y., Sakai A., Okada H., Watanabe R., Murakami S. (2013). MicroRNA-27a regulates lipid metabolism and inhibits hepatitis C virus replication in human hepatoma cells. J. Virol..

[27] Daneshpour M., Ghadimi-Daresajini A. (2023). Overview of miR-106a Regulatory Roles: From Cancer to Aging. Bioengineering.

[28] Gu J., Xu H., Chen Y., Li N., Hou X. (2022). MiR-223 as a Regulator and Therapeutic Target in Liver Diseases. Front. Immunol..

[29] Yang Y.-L., Huang Y.-H. (2025). MicroRNA-29a attenuates inflammation and fibrosis in an animal model of NASH through MCJ inhibition and hippo pathway regulation. Eur. J. Pharmacol..

[30] Wang L., Liu P., Han Y. (2024). Serum microRNA-181a Expression Level in Patients with Acute Liver Failure and Its Correlation with Prognosis. Int. J. Gen. Med..

[31] Shrivastava S., Petrone J., Steele R., Lauer G.M., Di Bisceglie A.M., Ray R.B. (2013). Up-regulation of Circulating miR-20a is correlated with Hepatitis C Virus-Mediated Liver Disease Progression. Hepatology.

[32] Patil R., Salunke P., Karpe Y.A. (2023). Unravelling the Tripartite Interactions Among Hepatitis E Virus RNA, miR-140 and hnRNP K. J. Mol. Biol..

[33] Yin W., Zhao Y., Ji Y.-J., Tong L.-P., Liu Y., He S.-X., Wang A.-Q. (2015). Serum/Plasma MicroRNAs as Biomarkers for HBV-Related Hepatocellular Carcinoma in China. BioMed Res. Int..

[34] Morimoto A., Kannari M., Tsuchida Y., Sasaki S., Saito C., Matsuta T., Maeda T., Akiyama M., Nakamura T., Sakaguchi M. (2017). An HNF4α-microRNA-194/192 signaling axis maintains hepatic cell function. J. Biol. Chem..

[35] Weseslindtner L., Macheleidt I., Eischeid H., Strassl R., Hofer H., Popow-Kraupp T., Dienes H.-P., Holzmann H., Odenthal M. (2016). Micro RNAs mir-106a, mir-122 and mir-197 are increased in severe acute viral hepatitis with coagulopathy. Liver Int..

[36] Majumdar S., Chowdhury D.R., Chakraborty B.C., Chowdhury A., Datta S., Banerjee S. (2025). MiR-451a attenuates hepatic steatosis and hepatitis C virus replication by targeting glycerol kinase. J. Transl. Med..

