# Supplementary material for: Longitudinal Follow-Up of microRNA Expression During Hepatitis E Virus Infection in Immunocompromised Hosts
Source: Biomolecules. 2026 May 28;16(6):799. doi: 10.3390/biom16060799 (PMC13297404; doi:10.3390/biom16060799)
Supplement: Supplementary file 1 [file biomolecules-16-00799-s001.zip › biomolecules-4260198-supplementary.pdf]

biomolecules

MDPI

Supplementary Materials

Table S1. Small RNAseq results in HEV-infected immunocompromised pigs and miRNAs considered for qPCR.

Name	Max group mean	Log_2 fold change	Fold change	*p*-value	FDR *p*-value	Bonferroni	Validation qPCR
ssc-miR-181a	324	−2.10013	−4.28749	1.29×10^{-6}	2.44×10^{-5}	0.000378	yes
ssc-miR-6529	1734.333333	−1.88377	−3.69037	7.45×10^{-8}	1.89×10^{-6}	2.19E-05	yes
ssc-miR-423-5p	42376.33333	−1.58478	−2.99961	8.56×10^{-6}	0.000139	0.002515	yes
ssc-miR-181b	188.6666667	−1.31741	−2.49219	0.00213	0.011845	0.626239	yes
ssc-miR-320	13028.5	−1.2921	−2.44884	6.75E-05	0.000733	0.019855	yes
ssc-miR-99a-5p	252.8333333	−1.10451	−2.15026	0.011796	0.042461	1	yes
ssc-miR-9841-3p	2551	−1.08889	−2.1271	0.002724	0.013801	0.800837	
ssc-miR-26a	1782	−1.02228	−2.03112	0.002193	0.011907	0.644845	
ssc-miR-378	187.5	−0.95886	−1.94378	0.001415	0.008717	0.415872	
ssc-miR-26b-5p	1046.833333	−0.92173	−1.89438	0.000367	0.002885	0.10789	
ssc-miR-125b	138.8333333	−0.7465	−1.67772	0.011914	0.042461	1	
ssc-miR-103	411.5	−0.74077	−1.67107	0.019979	0.063705	1	
ssc-miR-130b-3p	574.8333333	−0.60637	−1.52242	0.109097	0.245368	1	
ssc-let-7d-5p	2417.5	−0.5713	−1.48586	0.073442	0.19028	1	
ssc-let-7f-5p	7030.666667	−0.56107	−1.47536	0.085897	0.213766	1	
ssc-let-7a	18643.16667	−0.51603	−1.43001	0.104581	0.240852	1	
ssc-miR-30e-3p	122.3333333	−0.49627	−1.41056	0.082243	0.21069	1	
ssc-let-7c	22522	−0.49204	−1.40643	0.086546	0.213766	1	
ssc-miR-139-5p	663.3333333	−0.49161	−1.40601	0.147709	0.289157	1	
ssc-miR-339	2046.333333	−0.49061	−1.40504	0.153901	0.29315	1	
ssc-miR-126-3p	2089.166667	−0.46441	−1.37975	0.147494	0.289157	1	
ssc-let-7e	220.3333333	−0.43761	−1.35436	0.205867	0.358302	1	
ssc-miR-342	6348.166667	−0.36337	−1.28643	0.374316	0.493483	1	
ssc-miR-155-5p	699.3333333	−0.36306	−1.28615	0.301167	0.448893	1	
ssc-miR-28-3p	962.5	−0.36265	−1.28579	0.220729	0.372251	1	
ssc-miR-486	11122.16667	−0.28907	−1.22185	0.449068	0.559494	1	
ssc-miR-296-3p	955.5	−0.2817	−1.21563	0.324591	0.465452	1	
ssc-miR-423-3p	116.6666667	−0.26956	−1.20544	0.530874	0.630413	1	
ssc-miR-744	323	−0.2008	−1.14933	0.615014	0.701116	1	
ssc-miR-628-3p	112.3333333	−0.15556	−1.11385	0.634225	0.715858	1	
ssc-miR-1307	707.5	−0.09386	−1.06722	0.753856	0.809379	1	
ssc-miR-24-3p	1362.166667	−0.07023	−1.04989	0.786792	0.822884	1	
ssc-miR-151-5p	2062.666667	−0.02229	−1.01557	0.937697	0.941828	1	
ssc-miR-7134-3p	1296.333333	0.03002	1.021026	0.908105	0.920213	1	
ssc-miR-30d	4698.5	0.053266	1.037612	0.872093	0.891646	1	
ssc-miR-142-3p	423	0.06916	1.049106	0.849944	0.872915	1	
ssc-miR-98	794.6666667	0.074115	1.052715	0.811008	0.844337	1	
ssc-miR-126-5p	285.8333333	0.129589	1.093982	0.716511	0.787721	1	
ssc-miR-1306-5p	316.6666667	0.139325	1.10139	0.773948	0.818997	1	
ssc-miR-10a-5p	341.1666667	0.15949	1.116892	0.695326	0.769584	1	
ssc-miR-339-3p	245.5	0.168162	1.123626	0.614941	0.701116	1	
ssc-miR-223	729.5	0.189074	1.140031	0.565749	0.661492	1	
ssc-miR-130a	112.1666667	0.263672	1.200531	0.650829	0.726987	1	
ssc-miR-425-5p	603.6666667	0.297565	1.229068	0.354609	0.484136	1	

ssc-miR-150	1650	0.355572	1.279493	0.301231	0.448893	1	
ssc-miR-151-3p	174.5	0.518241	1.432208	0.111228	0.246214	1	
ssc-miR-335	255.8333333	0.550547	1.464641	0.128535	0.270261	1	
ssc-miR-2320-5p	344.6666667	0.583415	1.498391	0.147447	0.289157	1	
ssc-miR-185	2339.833333	0.656099	1.575816	0.058815	0.155928	1	
ssc-miR-10b	111.3333333	0.685559	1.608325	0.240904	0.381431	1	
ssc-miR-574-3p	151	0.718935	1.645967	0.036889	0.106463	1	
ssc-miR-142-5p	479.8333333	0.772892	1.708691	0.100292	0.238824	1	
ssc-miR-143-3p	102.6666667	0.80874	1.751681	0.237433	0.38123	1	
ssc-miR-22-3p	194.8333333	0.850536	1.803171	0.0112	0.042461	1	
ssc-miR-128	341.8333333	0.862614	1.81833	0.092486	0.224329	1	
ssc-miR-30a-5p	227	0.872883	1.831318	0.008776	0.035105	1	Yes*
ssc-miR-7-5p	499.3333333	0.927921	1.902533	0.033789	0.098769	1	
ssc-miR-361-5p	322.6666667	0.967885	1.95597	0.011372	0.042461	1	
ssc-let-7d-3p	99.33333333	1.018899	2.026372	0.039907	0.113734	1	
ssc-let-7g	11676.33333	1.062152	2.088044	0.001414	0.008717	0.415717	
ssc-miR-186-5p	162	1.152438	2.222892	0.008079	0.033493	1	
ssc-miR-30e-5p	2272.166667	1.2067	2.308091	4.29E-05	0.000515	0.012607	
ssc-miR-210	113.3333333	1.250014	2.378437	0.045196	0.124153	1	
ssc-miR-17-5p	178.5	1.286058	2.438608	0.000155	0.001536	0.045561	
ssc-miR-193a-5p	730	1.29637	2.456102	0.000223	0.002036	0.065649	
ssc-miR-339-5p	436.6666667	1.305993	2.472539	0.001936	0.011035	0.569179	
ssc-miR-425-3p	248.8333333	1.312767	2.484175	0.000127	0.001313	0.037259	
ssc-miR-21-5p	158.5	1.359267	2.565547	0.000317	0.002777	0.093115	
ssc-miR-92a	2687.833333	1.404009	2.646359	1.45×10^{-5}	0.000214	0.004255	
ssc-miR-15b	495.8333333	1.40859	2.654776	0.002529	0.013103	0.743415	
ssc-miR-191	42428.16667	1.427412	2.689637	2.57×10^{-5}	0.000345	0.007567	yes
ssc-miR-1842	385.3333333	1.541482	2.910934	0.00019	0.001807	0.055936	Yes*
ssc-miR-532-5p	253.6666667	1.626727	3.088116	6.26×10^{-5}	0.000714	0.018414	Yes*
ssc-miR-27a	111.1666667	1.685971	3.217569	0.013878	0.047228	1	yes
ssc-miR-20a-5p	301.5	1.907087	3.75051	1.5×10^{-5}	0.000214	0.004418	yes
ssc-miR-23a-3p	601.1666667	1.9574	3.883615	3.16×10^{-5}	0.000401	0.009301	yes
ssc-miR-149	229	1.992743	3.97993	1.33×10^{-7}	3.04×10^{-6}	3.92×10^{-5}	Yes*
ssc-miR-29a-3p	627.5	2.078055	4.222376	2.87×10^{-7}	5.95×10^{-6}	8.44×10^{-5}	yes
ssc-miR-16	55113.5	2.189317	4.560895	3.52×10^{-11}	1.15×10^{-9}	1.03×10^{-8}	yes
ssc-miR-140-3p	1988	2.206798	4.616494	3.93×10^{-14}	2.98×10^{-12}	1.15×10^{-11}	yes
ssc-miR-574-3p	770.5	2.310041	4.958971	4.09×10^{-12}	1.87×10^{-10}	1.2×10^{-9}	yes
ssc-let-7i-5p	1582.5	2.588632	6.01528	8.12×10^{-14}	4.63×10^{-12}	2.39×10^{-11}	Yes*
ssc-miR-660	226.3333333	3.142422	8.830054	2.5×10^{-11}	9.51×10^{-10}	7.36×10^{-9}	yes
ssc-miR-451a	636	3.741582	13.37607	1.01×10^{-10}	2.87×10^{-9}	2.96×10^{-8}	yes
ssc-miR-122-5p	14159.5	5.387356	41.85581	1.85×10^{-33}	4.22×10^{-31}	5.44×10^{-31}	Yes*

Results correspond to samples collected 10 weeks post infection (*, miRNAs excluded after qPCR analysis due to low signal or amplification issues). Of note, ssc-miR-192-5p, which does not appear in this lit, was also considered for qPCR quantification based on the literature [2,34].

Table S2. Correlation between HEV viremia (log UI/mL) in pigs and miRNA expression (ΔCt value). The grey columns indicate the *p*-values.

microRNAs overtime	4 weeks p.i.		10 weeks p.i.		11 weeks p.i.		10 to 11 weeks p.i.		4 to 10 weeks p.i.		4 to 11 weeks p.i.	
**microRNAs**	**Pearson r**	***p*-value**	**Pearson r**	***p*-value**	**Pear-sonr**	***p*-value**	**Fisher r-to-z**	***p*_value**	**Fisher r-to-z**	***p*_value**	**Fisher r-to-z**	***p*_value**
**ssc-miR-16**	0.497	0.503	0.041	0.938	−0.559	0.249	0.824	0.205	0.436	0.331	1.019	0.154
**ssc-miR-20a-5p**	0.457	0.543	0.035	0.948	−0.304	0.558	0.427	0.335	0.397	0.346	0.699	0.242
**ssc-miR-23a-3p**	0.571	0.429	−0.189	0.720	−0.600	0.208	0.616	0.269	0.727	0.234	1.162	0.123
**ssc-miR-27a**	0.656	0.344	0.084	0.874	−0.600	0.208	0.952	0.171	0.607	0.272	1.28	0.100
**ssc-miR-29a-3p**	0.634	0.366	−0.618	0.191	0.687	0.132	**−1.915**	**0.028***	1.274	0.101	−0.081	0.468
**ssc-miR-30d**	0.621	0.379	0.439	0.384	0.108	0.838	0.443	0.329	0.222	0.412	0.535	0.296
**ssc-miR-99a-5p**	0.654	0.346	−0.215	0.682	−0.129	0.808	−0.109	0.457	0.867	0.193	0.79	0.215
**ssc-miR-140-3p**	0.636	0.364	−0.508	0.303	0.118	0.824	−0.832	0.203	1.136	0.128	0.548	0.292
**ssc-miR-181a**	0.654	0.346	−0.301	0.562	0.287	0.581	−0.742	0.229	0.947	0.172	0.422	0.337
**ssc-miR-181b**	0.381	0.619	0.289	0.579	0.449	0.372	0.330	0.371	0.09	0.464	−0.07	0.472
**ssc-miR-191**	0.802	0.198	0.102	0.847	−0.257	0.624	0.447	0.327	0.868	0.193	1.185	0.118
**ssc-miR-192-5p**	0.621	0.379	0.327	0.527	0.384	0.453	−0.080	0.468	0.335	0.369	0.279	0.390
**ssc-miR-320**	0.675	0.326	0.450	0.370	−0.505	0.307	1.275	0.101	0.289	0.386	1.191	0.117
**ssc-miR-423-5p**	0.810	0.190	−0.506	0.305	**0.914**	**0.011***	**−2.582**	**0.005****	1.46	0.072	−0.366	0.357
**ssc-miR-425-5p**	0.6302	0.370	0.019	0.971	−0.209	0.691	0.283	0.389	0.626	0.266	0.826	0.204
**ssc-miR-451a**	0.785	0.215	−0.083	0.876	−0.685	0.133	0.926	0.177	0.989	0.161	**1.643**	**0.050***
**ssc-miR-574-3p**	−0.042	0.958	−0.280	0.591	0.449	0.372	−0.943	0.173	0.212	0.416	−0.455	0.325
**ssc-miR-660**	−0.574	0.427	−0.402	0.430	0.449	0.372	−1.113	0.133	−0.197	0.422	−0.984	0.163
**ssc-miR-6529**	−0.502	0.498	0.466	0.352	0.449	0.372	0.027	0.489	−0.916	0.180	−0.897	0.185

In bold, results with $p < 0.05$. Asterisks show *p*-value <0.05 (*) and *p*-value <0.01(**).

Table S3. human sera samples selected for miRNA analysis.

Group	Patient	Sample	weeks post diagnosis	Early/ Late/ cure sample	HEV RNA IU/ml	ALT IU/L	Tacrolimus trough concentration ng/mL
Acute	P3	P3-1	5	early	2.06×10^5	138	10
		P3-2	10	early	3.93×10^5	145	6.5
		P3-3	154	cure	0	18	5.5
	P30	P30-1	0	early	1.48×10^6	97	5
		P30-2	35	cure	0		
	P53	P53-1	0	early	9.03×10^5	195	
		P53-2	11	early	4.80×10^5	106	6.1
		P53-3	37	cure	0	42	6.2
	P50	P50-1	0	early	3.13×10^8	478	
		P50-2	2	early	1.89×10^7	373	
		P50-3	9	cure	0	18	
Chronic	C16	C16-1	25	late	1.66×10^2	20	6.5
		C16-2	100	late	2.05×10^3	42	4.9
		C16-4	296	cure	0	53	
	C9	C9-1	7	early	3.55×10^4	21	6.3
		C9-2	24	late	8.05×10^5	22	9.9
		C9-4	443	Cure	0	11	5.8
	P20	P20-1	0	early	1.14×10^3	36	5.6
		P20-3	17	late	6.93×10^5	58	4.2
		P20-4	205	cure	0	12	9
	C10	C10-1	0	early	1.06×10^5	54	8.5
		C10-3	24	late	2.65×10^4	54	4.8
		C10-5	207	cure	0	51	
	C18	C18-1	0	early	9.40×10^7	134	6.9
		C18-2	15	late	8.91×10^7	58	3.8
		C18-3	27	cure	0	8	4.4

Table S4. Human miRNAs considered for qPCR.

N°	microRNA	*Code*	Reference
**1**	hsa-miR-122-5p	*YP00205664*	[1,2]
**2**	hsa-miR-194-5p	*YP00204080*	[1]
**3**	hsa-miR-885-5p*	*YP00204473*	[1]
**4**	hsa-miR-30a-5p	*YP00205695*	[1]
**5**	hsa-miR-221-3p	*YP00204532*	[1]
**6**	hsa-miR-223-3p	*YP00205986*	[1]
**7**	hsa-miR-27a-3p	*YP00206038*	[1]
**8**	hsa-miR-335-5p	*YP02119293*	[1]
**9**	hsa-miR-140-5p*	*YP00204540*	[32]
**10**	hsa-miR-512-3p*	*YP00204382*	[19]
**11**	hsa-miR-214-3p*	*YP00204510*	[5]
**12**	hsa-miR-99a-5p	*YP00204521*	[2]
**13**	hsa-miR-125b-5p	*YP00205713*	[2]
**14**	hsa-miR-192-5p	*YP00204099*	[2]
**15**	hsa-miR-590-5p*	*YP00204222*	[20]
**16**	hsa-miR-526b-3p*	*YP00205984*	[20]
**17**	hsa-miR-151-3p*	*YP00204576*	[20]
**18**	hsa-miR-181a-5p	*YP00206081*	[30]

**19**	hsa-miR-628-3p*	*YP00206057*	[20]
**20**	hsa-miR-106a-5p	*YP00204563*	[35]
**21**	miR-451a	*YP02119305*	[36]
**22**	miR-23a-3p	*YP00204772*	[1]
**23**	hsa-miR-222-3p	*YP00204551*	Control
**24**	hsa-let-7a-5p	*YP00205727*	Control
**25**	hsa-let-7b-5p	*YP00204750*	Control
**26**	hsa-let-7i-5p	*YP00204394*	Control
**27**	hsa-miR-425-5p	*YP00204337*	Control
**28**	hsa-miR-30e-5p	*YP00204714*	Control

*, indicates miRNA which could not be amplified.

Table S5. Correlation between HEV viremia (log UI/mL) in patients and miRNA expression (ΔCt value).

**Correlation between microRNAs and HEV viremia**	**early_resolving (*n* = 4)**		**early_chronic (*n* = 4)**		**Late_chronic (*n* = 5)**		**Early to late in chronic patients**	
**microRNAs**	**Pearson r**	***p*-value**	**Pearson r**	***p*-value**	**Pearson r**	***p*-value**	**Fisher r-to-z**	***p*-value**
**miR-23a-3p**	0.894	0.106	−0.442	0.558	−0.539	0.349	0.105	0.458
**hsa-miR-27a-3p**	0.766	0.235	−0.774	0.226	−0.175	0.779	−0.698	0.243
**hsa-miR-30a-5p**	−0.539	0.461	−0.478	0.522	0.415	0.488	−0.785	0.216
**hsa-miR-30e-5p**	−0.113	0.887	−0.395	0.605	−0.429	0.471	0.033	0.487
**hsa-miR-99a-5p**	−0.573	0.427	−0.507	0.493	−0.550	0.337	0.048	0.481
**hsa-miR-106a-5p**	0.775	0.225	−0.363	0.637	−0.306	0.617	−0.053	0.479
**hsa-miR-122-5p**	−0.439	0.561	−0.862	0.138	−0.443	0.455	−0.674	0.250
**hsa-miR-125b-5p**	−0.387	0.613	−0.738	0.262	0.168	0.787	−0.912	0.181
**hsa-miR-181a-5p**	0.523	0.477	−0.315	0.685	**−0.965**	**0.008****	1.375	0.085
**hsa-miR-192-5p**	−0.113	0.887	−0.672	0.328	−0.344	0.571	−0.372	0.355
**hsa-miR-194-5p**	−0.681	0.319	0.069	0.931	0.242	0.696	−0.144	0.443
**hsa-miR-221-3p**	0.720	0.280	−0.340	0.660	−0.008	0.990	−0.283	0.389
**hsa-miR-222-3p**	0.111	0.889	−0.926	0.074	0.415	0.488	**−1.690**	**0.046***
**hsa-miR-223-3p**	0.711	0.289	−0.472	0.529	0.000	1.000	−0.418	0.338
**hsa-miR-335-5p**	0.083	0.917	−0.044	0.956	−0.606	0.279	0.538	0.295
**hsa-miR-425-5p**	0.255	0.745	−0.424	0.576	**−0.964**	**0.008****	1.263	0.103
**miR-451a**	0.451	0.549	−0.367	0.633	−0.640	0.245	0.304	0.380
**hsa-let-7a-5p**	0.713	0.287	−0.308	0.692	−0.712	0.177	0.467	0.320
**hsa-let-7b-5p**	0.454	0.547	−0.547	0.453	**−0.935**	**0.02***	0.885	0.188
**hsa-let-7i-5p**	−0.131	0.869	−0.340	0.660	−0.802	0.102	0.613	0.270

In bold, results with $p<0.05$. Asterisks show *p*-value <0.05 (*) and *p*-value <0.01(**).

Table S6. Correlation between ALT and miRNA expression (ΔCt value) in patients.

**Correlation between miRNA and ALT**	**early_resolving (*n* = 4)**		**early_chronic (*n* = 4)**		**Late_chronic (*n* = 5)**		**Early_chronic to late_chronic**	
**microRNAs**	**Pearson r**	***p*-value**	**Pearson r**	***p*-value**	**Pearson r**	***p*-value**	**Fisher r-to-z**	***p*-value**
**hsa-miR-122-5p**	−0.480	0.520	−0.711	0.290	−0.469	0.425	−0.311	0.378
**hsa-miR-194-5p**	−0.755	0.245	0.132	0.868	0.297	0.628	−0.142	0.444
**hsa-miR-30a-5p**	−0.667	0.333	−0.528	0.472	−0.024	0.970	−0.46	0.323
**hsa-miR-221-3p**	0.775	0.225	−0.332	0.668	−0.875	0.052	0.824	0.205
**hsa-miR-223-3p**	0.710	0.290	−0.632	0.368	−0.679	0.208	0.067	0.473
**hsa-miR-27a-3p**	0.693	0.307	−0.822	0.179	**−0.884**	**0.0464**	0.188	0.425
**hsa-miR-335-5p**	0.049	0.951	0.148	0.852	−0.734	0.158	0.887	0.188

**hsa-miR-99a-5p**	−0.650	0.350	−0.449	0.551	−0.433	0.466	−0.016	0.494
**hsa-miR-125b-5p**	−0.439	0.561	−0.616	0.384	0.272	0.658	−0.815	0.208
**hsa-miR-192-5p**	−0.207	0.793	−0.588	0.412	0.017	0.978	−0.565	0.286
**hsa-miR-181a-5p**	0.650	0.351	−0.063	0.937	−0.503	0.388	0.4	0.344
**hsa-miR-106a-5p**	0.780	0.220	−0.508	0.492	**−0.920**	**0.0268**	0.84	0.2
**miR-451a**	0.325	0.675	−0.602	0.398	−0.304	0.619	−0.312	0.377
**miR-23a-3p**	0.935	0.065	−0.547	0.453	−0.789	0.113	0.371	0.355
**hsa-miR-222-3p**	0.211	0.789	−0.870	0.130	−0.024	0.970	−1.069	0.143
**hsa-let-7a-5p**	0.814	0.186	−0.046	0.954	−0.316	0.605	0.23	0.409
**hsa-let-7b-5p**	0.434	0.566	−0.336	0.664	−0.371	0.539	0.033	0.487
**hsa-let-7i-5p**	0.026	0.974	−0.468	0.532	−0.528	0.361	0.065	0.474
**hsa-miR-425-5p**	0.107	0.893	−0.522	0.478	−0.229	0.710	−0.282	0.389
**hsa-miR-30e-5p**	−0.261	0.739	−0.605	0.395	−0.152	0.807	−0.447	0.327

In bold, results with p<0.05.

Table S7. Correlation between tacrolimus trough concentration and miRNA expression (ΔCt value) in patients.

**Correlation between miRNA and tacrolimus**	**early_resolving (*n* = 4)**		**early_chronic (*n* = 4)**		**Late_chronic (*n* = 5)**		**Early_chronic to late_chronic**	
**microRNAs**	**Pearson r**	***p*-value**	**Pearson r**	***p*-value**	**Pearson r**	***p*-value**	**Fisher r-to-z**	***p*-value**
**hsa-miR-122-5p**	−0.679	0.321	0.457	0.543	0.056	0.929	0.357	0.36
**hsa-miR-194-5p**	−0.518	0.482	0.815	0.185	−0.564	0.323	1.454	0.073
**hsa-miR-30a-5p**	−0.475	0.525	0.336	0.664	−0.134	0.830	0.396	0.346
**hsa-miR-221-3p**	−0.349	0.651	0.584	0.417	0.809	0.097	−0.372	0.355
**hsa-miR-223-3p**	−0.668	0.332	−0.146	0.854	0.485	0.408	−0.552	0.29
**hsa-miR-27a-3p**	−0.826	0.174	0.122	0.879	0.839	0.076	−0.894	0.186
**hsa-miR-335-5p**	−0.855	0.145	**0.994**	**0.0062****	0.644	0.241	**1.746**	**0.04***
**hsa-miR-99a-5p**	−0.632	0.368	0.644	0.356	0.274	0.656	0.395	0.346
**hsa-miR-125b-5p**	−0.733	0.267	0.612	0.389	−0.608	0.277	1.158	0.123
**hsa-miR-192-5p**	−0.922	0.078	0.599	0.401	−0.283	0.644	0.802	0.211
**hsa-miR-181a-5p**	0.537	0.463	0.863	0.137	0.364	0.547	0.754	0.225
**hsa-miR-106a-5p**	−0.602	0.398	0.005	0.995	**0.934**	**0.0201***	−1.375	0.085
**miR-451a**	**−0.951**	**0.0487***	−0.749	0.251	0.619	0.266	−1.383	0.083
**miR-23a-3p**	−0.254	0.746	0.139	0.861	0.773	0.125	−0.725	0.234
**hsa-miR-222-3p**	0.925	0.075	0.264	0.736	−0.134	0.830	0.331	0.37
**hsa-let-7a-5p**	0.118	0.882	0.797	0.203	0.006	0.992	0.885	0.188
**hsa-let-7b-5p**	−0.817	0.183	0.803	0.197	0.436	0.464	0.522	0.301
**hsa-let-7i-5p**	0.486	0.514	0.093	0.907	0.743	0.151	−0.705	0.24
**hsa-miR-425-5p**	−0.901	0.100	0.178	0.822	0.179	0.773	−0.001	0.5
**hsa-miR-30e-5p**	−0.813	0.187	−0.411	0.589	0.304	0.620	−0.613	0.27

In bold, results with p<0.05. Asterisks show p-value <0.05 (*) and p-value <0.01(**).

Table S8. Correlation between relevant clinical parameters in patients with HEV infection. Asterisks show *p*-value <0.05 (*).

Correlation between relevant clinical parameters	early_resolving (*n* = 4)		early_chronic (*n* = 4)		Late_chronic (*n* = 5)		early_chronic to Late_chronic	
	**Pearson r**	***p*-value**	**Pearson r**	***p*-value**	**Pearson r**	***p*-value**	**Fisher r-to-z**	***p*-value**
**ALT IU/L & Tacrolimus trough concentration ng/mL**	−0.167	0.833	0.243	0.757	**−0.894**	**0.0411***	1.379	0.084
**HEV RNA log UI/mL & Tacrolimus trough concentration ng/mL**	−0.273	0.727	0.041	0.959	−0.422	0.479	0.401	0.344
**HEV RNA log UI/mL & ALT IU/L**	**0.987**	**0.0131***	**0.964**	**0.0364***	0.442	0.456	1.241	0.107

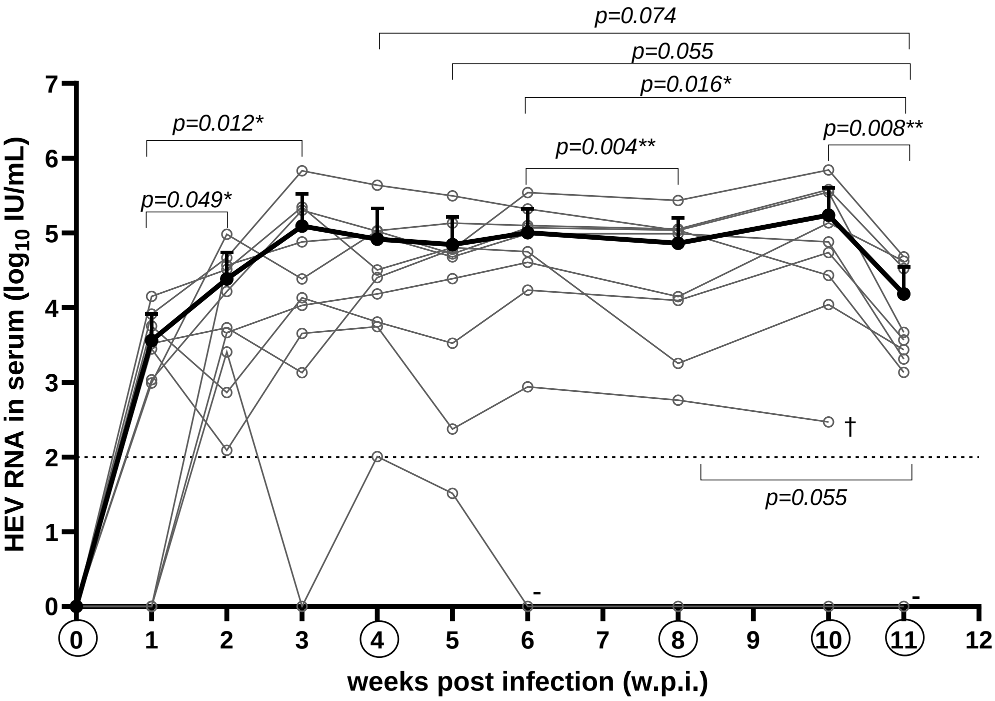

Figure S1. Quantification of HEV RNA in sera during 11 weeks of infection in immunocompromised pigs (modified from [6]). Circles on the X-axis (A) indicate the weeks from which the pig serum samples were extracted for this study. Asterisks show *p*-value <0.05 (*) and *p*-value <0.01(**).

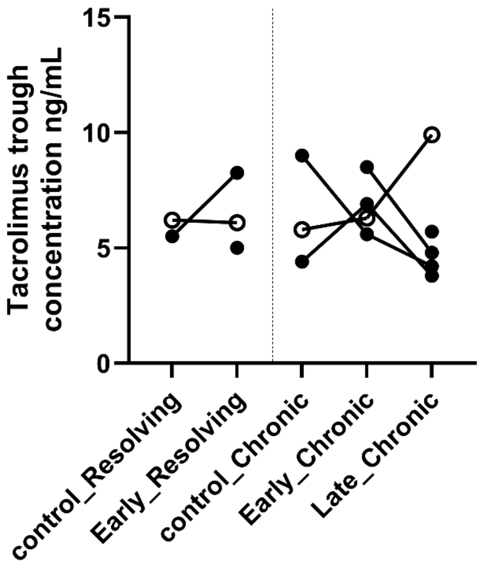

Figure S2. Individual follow-up of tacrolimus trough concentration in HEV-infected immunocompromised patients. Full circles, Kidney transplant; Empty circles, heart transplant.
